# Bridging the gap with multispecific immune cell engagers in cancer and infectious diseases

**DOI:** 10.1038/s41423-024-01176-4

**Published:** 2024-05-24

**Authors:** Camille Rolin, Jacques Zimmer, Carole Seguin-Devaux

**Affiliations:** 1 Department of Infection and Immunity, Luxembourg Institute of Health, 29 Rue Henri Koch, L-4354 Esch-Sur-Alzette, Luxembourg; 2 University of Luxembourg, 2 Place de l’Université, L-4365 Esch-sur-Alzette, Luxembourg

**Keywords:** Immune cell engagers, Multispecific antibodies, Cancer, Virus, Immunotherapy, Immunotherapy, Infectious diseases, Cancer therapy

## Abstract

By binding to multiple antigens simultaneously, multispecific antibodies are expected to substantially improve both the activity and long-term efficacy of antibody-based immunotherapy. Immune cell engagers, a subclass of antibody-based constructs, consist of engineered structures designed to bridge immune effector cells to their target, thereby redirecting the immune response toward the tumor cells or infected cells. The increasing number of recent clinical trials evaluating immune cell engagers reflects the important role of these molecules in new therapeutic approaches for cancer and infections. In this review, we discuss how different immune cell types (T and natural killer lymphocytes, as well as myeloid cells) can be bound by immune cell engagers in immunotherapy for cancer and infectious diseases. Furthermore, we explore the preclinical and clinical advancements of these constructs, and we discuss the challenges in translating the current knowledge from cancer to the virology field. Finally, we speculate on the promising future directions that immune cell engagers may take in cancer treatment and antiviral therapy.

## Introduction

For more than 100 years, immunotherapy has slowly emerged as a revolutionary treatment for many diseases, most predominantly cancer. The story of immunotherapy started in the 19th century with “Coley’s toxin”, a combination of heat-inactivated bacteria injected into cancer patients after surgery to induce an immune response and ultimately anticancer effects [1, 2]. The approach was based on prior observations that postoperative wound infections, a common adverse event of surgery, had previously resulted in the complete regression of a patient’s inoperable neck sarcoma [2].

The field has largely evolved since then, and the discovery and understanding of cytokines, as well as effector immune cells, has allowed immunotherapy to become a major treatment option not only for cancer [1] but also for other diseases, including infections (bacterial and viral), allergies, and inflammatory and autoimmune pathologies [3].

Immunotherapy is currently defined as “a therapeutic approach that targets or manipulates the immune system” [4]. More specifically, it regroups different options into two main subgroups—active and passive therapy—based not only on the therapeutic agent used but also on the immune competence of the recipient. While active immunotherapy harnesses immune effector cells in immunocompetent patients, passive immunotherapy approach aims at compensating for the defective immune system of the recipients through the administration of exogenous molecules or effector cells [4]. Overall, immunotherapy notably includes cancer vaccines, cytokines, therapeutic antibodies, oncolytic viruses, adoptive cellular transfer and, more recently, chimeric antigen receptor (CAR) therapy. These various approaches have been widely described and reviewed [5, 6].

Despite comprehensive coverage in the literature, a specific branch within antibody-based therapy, namely, immune cell engagers, has received, until very recently [5], comparatively limited attention. Immune cell engagers are engineered antibody-based structures designed to bridge immune cells to their target, thus redirecting the immune effector response toward the bound cell pathogen. While they offer more targeting combinations, as well as enhanced specificity, their actions are mainly derived from the specific features of monoclonal antibodies (mAbs).

**Fig. 1:**
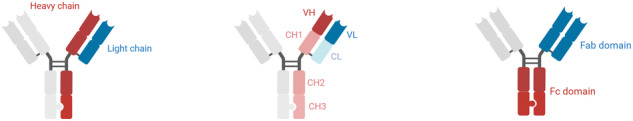
Structures of monoclonal antibodies. VH variable heavy chain, VL variable light chain, CH constant heavy chain, CL constant light chain, Fab fragment antigen binding, Fc fragment crystallizable. Created with Biorender

Monoclonal antibodies have become crucial tools in biomedical research against various human diseases [6]. They are structurally composed of two identical heterodimers, each containing a heavy and a light chain. Each of the light chains possesses one variable domain (VL) and one constant domain (CL), and each of the heavy chains contains three constant domains (CH) and one variable domain (VH) (Fig. 1) [7]. On the one hand, the combination of the variable domains (VL and VH), the constant domain of the light chain (CL) and the first constant domain of the heavy chain (CH1) constitute the antigen-binding site (Fab), an indispensable part allowing mAbs to bind to their specific target. On the other hand, the C-terminal constant fragments of the heavy chain encompass the fragment crystallizable region (Fc), which is extremely important for the functional effector role of antibodies in immune cells [7].

The structure of monospecific antibodies has been further improved to multispecific antibodies, defined as antibody-based structures possessing at least two different antigen-binding sites [8]. These constructs were designed to overcome treatment resistance observed with conventional monospecific constructs by tackling various targets simultaneously (e.g., immune escape pathways at the surface of cancer cells) [6]. The identification of the binding of immune cells to cancer cells early in the 1960s paved the way for the development of immune cell engagers and the field of bispecific antibodies in general, which are now represented by approximately 100 compounds [8]. Among these compounds, almost 30 are currently being evaluated in clinical trials, and some have even been approved for clinical use [8, 9].

For a long time, research on immune cell engagers has focused almost exclusively on T lymphocytes in cancer treatment, probably because of the extremely important role of these cells in the anticancer immune response [10]. However, with time, the understanding of the roles of other immune cells, including natural killer (NK) cells, myeloid cells such as macrophages and neutrophils, dendritic cells and other cell types such as γδT cells and mucosal-associated invariant T (MAIT) cells, has grown. The potential of immune cell engagers was also enhanced by combination with complementary approaches, such as cytokine therapy, making them important new agents in immunotherapy, especially in cancer and human immunodeficiency virus (HIV) or hepatitis B virus (HBV) infection. Interestingly, both cancer and antiviral therapies face common challenges, and tackling them would help advance research on both diseases [11], although viral diseases such as HIV face their own problems due to the persistence of viral reservoirs. Indeed, HIV-1 escapes immune recognition by hiding in quiescent cells such as memory CD4+ T cells, plasmacytoid DCs (pDCs) and macrophages that trigger a persistent low level of replication, resulting in immune dysfunction, comorbidities and chronic inflammation.

Consistently, this review aims to provide a thorough exploration of immune cell engagers, elucidating their mechanisms and elaborating on their promising applications within the dynamic landscape of immunotherapy against cancer and infectious diseases. In light of the recently published exhaustive review by the Vivier group about new cell engagers in cancer immunotherapy [5], we will focus more on other applications, predominantly in the virology field.

Although a large proportion of the available literature concerns T cells and NK cells, we will also introduce the early achievements in the less studied but nevertheless encouraging myeloid cell engagers. The goal of this review is to depict the wide spectrum of multispecific antibodies engaging immune cells regarding both the cell types they can target and the diseases they tackle.

## Multispecific engagers binding T cells in cancer immunotherapy

T lymphocytes are CD3^+^ immune cells involved in the activation and regulation of the immune system. Because of their central action and their ability to establish immune memory, these cells have quickly emerged as the main actors in immunotherapy for several diseases, including cancer and HIV. Many approaches have been developed to stimulate T cells to fight their targets. First developed in the field of cancer therapy, these approaches include the administration of the cytokine interleukin (IL)-2, checkpoint inhibitors and, more recently, CAR-T cells (i.e., T cells with genetically engineered TCRs), which hold great potential and are currently used in the clinic to treat different cancers, such as B-cell malignancies [12].

However, many challenges remain for manufacturing these engineered T cells and, more importantly, for their limited efficacy and unsatisfactory toxicity profile [13]. Different aspects must be addressed to overcome these issues, such as access to antigens, chronic immune dysfunction in patients or the presence of a hostile microenvironment. Multispecific immune engagers could be key actors in overcoming some of these limitations, as they exhibit improved pharmacokinetic and pharmacodynamic properties, as well as enhanced cytotoxicity against HIV-infected and tumor cells [14, 15].

### Bispecific T-cell engagers (BiTEs)

The first immune engager developed was the bispecific T-cell engager (BiTE), which can cross-link T cells and cancer cells. By binding to specific antigens expressed on the surface of both cell types, BiTEs bring the effector and the tumor cells closer and therefore facilitate the formation of immunological synapses [16]. Ultimately, this process enhances the cytotoxicity of T cells toward their target through the formation of membrane pores via the perforin-granzyme pathway, which leads to the lysis of cancer cells [13, 17, 18].

The first BiTE approved by the FDA and EMA was blinatumomab, an anti-CD19 × anti-CD3 compound intended for the treatment of B-cell acute lymphoblastic leukemia (B-ALL) [16]. Blinatumomab has since paved the way for the development of many other BiTEs, as represented by the 100 ongoing clinical trials currently evaluating such molecules in oncology, both for the treatment of liquid and solid tumors [12, 17]. In 2022, three bispecific T-cell engagers were approved in Europe and the USA: tebentafusp (anti-CD3 × anti-gp100 compound for the treatment of metastatic uveal melanoma), mosunetuzumab (anti-CD3 × anti-CD20 compound for the treatment of follicular lymphoma), and teclistamab (anti-CD3 x anti-BCMA compound against multiple myeloma) [19]. Moreover, the “Annual Antibodies to Watch article series 2023” reported 3 BiTEs in late-stage clinical studies that were very likely to receive approval from the FDA and EMA in 2023 (the anti-CD3 × anti-CD20 compound odronextamab, the anti-CD3 × anti-BCMA compound linvoseltamab and the anti-CD3 × anti-GPRC5D compound talquetamab) [19]. According to the very recent 2024 report of this series, odronextamab and linvoseltamab are still under evaluation by regulatory authorities, while talquetamab has received FDA approval and EMA conditional authorization [20].

### Tri- and tetra-specific T-cell engagers

Despite great initial enthusiasm, BiTEs do not always reach sufficient efficiency, as they target only one receptor on the surface of T cells. However, treatments targeting two T-cell receptors (such as CD3 and the costimulator CD28) through the development of tri- or tetraspecific T-cell engagers could provide the costimulatory signal necessary for proper T-cell function.

Wu and collaborators designed an anti-CD3 × anti-CD28 × anti-CD38 engager (SAR442257) that targets myeloma cells and efficiently activates T cells [21]. In preclinical studies, SAR442257 was shown to suppress myeloma growth in humanized mice and to stimulate the proliferation of memory/effector T cells while being well tolerated in NHPs. This compound consequently became the first trispecific T-cell engager to enter a phase I clinical trial in May 2020 for the treatment of multiple myeloma and non-Hodgkin’s lymphoma (NCT04401020) [22].

The first-in-class tetrafunctional T-cell engager was reported in 2022, in which a fourth arm containing an anti-IL-6 receptor (IL-6R) was added to an anti-EGFR x anti-PDL1 × anti-CD3 agent to modulate the activity of this cytokine and decrease cytokine release syndrome [23]. The authors showed attenuated IFN-γ production while maintaining sufficient T-cell-mediated cytotoxicity against cancer cells. This new strategy has the ability to modulate cytokine storms, an unfortunate side effect often observed with T-cell immunotherapy, although the release of other proinflammatory cytokines still needs further investigation [23].

### Alternative formats

Rather than targeting two T-cell antigens, Tapia-Galisteo et al. developed a trispecific T-cell engager (TriTE) that targets two tumor-associated antigens (TAAs), EpCAM and EGFR, to increase tumor selectivity and sensitivity in the case of immune escape via antigen loss [16]. Although this study provides proof-of-concept, better in vivo therapeutic outcomes were described compared to bispecific anti-CD3 x anti-EGFR antibodies [16].

**Fig. 2:**
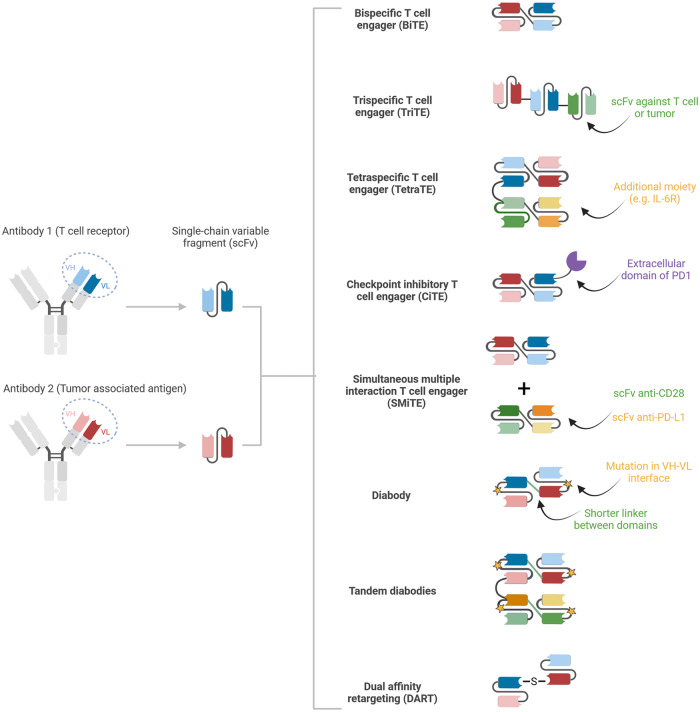
Various T-cell engager (TCE) formats described in immuno-oncology. Single-chain variable fragments (scFvs) derived from T-cell receptor antibodies (blue) and from tumor-associated antigen antibodies (red) can associate to form bispecific T-cell engagers (BiTEs). BiTEs can be combined with a third scFv (green) to form a trispecific T-cell engager (TriTE) and with an additional moiety to form a tetraspecific T-cell engager (TetraTE). BiTEs can also be complemented with the extracellular domain of PD1 to form a checkpoint inhibitor T-cell engager (CiTE) or administered with a second molecule, such as an anti-CD28 × anti-PDL1, as in the simultaneous multiple interaction T-cell engager (SMiTE). Various molecular modifications, such as mutations of the VH-VL interface, shorter links between domains and the addition of disulfide bonds, have led to the development of additional formats, such as diabody, tandem diabody and dual affinity retargeting (DART). Created with Biorender

Other T-cell engagers have been developed, such as bifunctional checkpoint inhibitory T-cell engaging (CiTE) and simultaneous multiple interaction T-cell engaging (SMiTE) antibodies [18]. On the one hand, CiTEs aim to fight resistance to classical BiTE therapy acquired via the upregulation of immune checkpoint inhibitors (mostly PD-1/PD-L1) by adding a low-affinity extracellular domain of PD-1, thereby decreasing the binding of PD-1 to PD-L1. On the other hand, SMiTEs consist of a pair of BiTEs, namely, an anti-CD3 × TAA and an anti-CD28 × anti-PD-L1, to combine proper T-cell activation and inhibition of resistance mechanisms [18]. Other alternatives include diabodies (molecules similar to BiTEs but with a decreased linker size and modified structure to increase the heterodimerization and stability of the construct), tandem diabodies (two diabodies in tandem to double the number of antigen sites and therefore increase the pharmacodynamic and pharmacokinetic properties), and dual affinity retargeting (DART) proteins (two polypeptide chains linked by a disulfide bond for enhanced stability) [7]. This list, although nonexhaustive, is depicted in Fig. 2, highlighting the extremely large range of possibilities to engage T cells and cancer cells and undoubtedly the importance of T-cell engagers in cancer immunotherapy.

## Extending T-cell engagers to infectious diseases

**Fig. 3:**
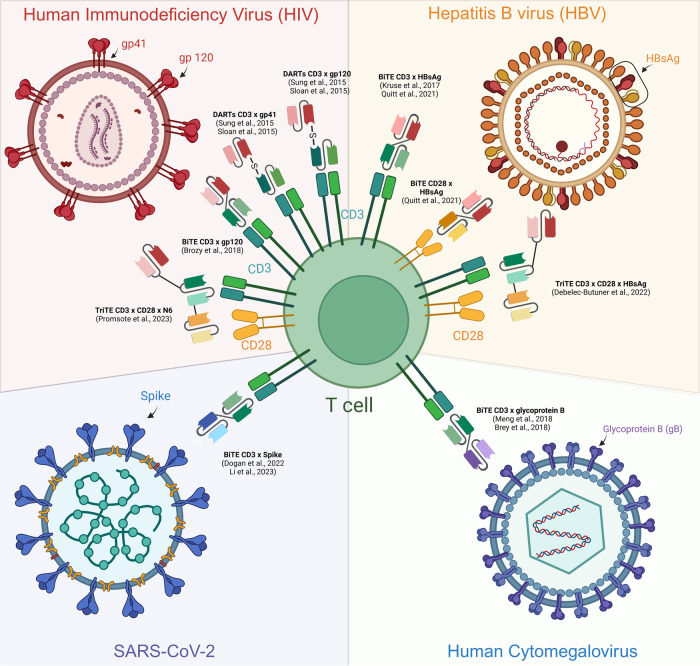
T-cell engagers described in viral diseases. DART dual affinity retargeting, HBSAg hepatitis B surface antigen. Created with Biorender

Driven by the success of these constructs in cancer immunotherapy, bispecific T-cell engagers are also being developed for the treatment of infectious diseases (Fig. 3).

Among these, HIV has attracted the most interest. Human immunodeficiency virus is transmitted through blood and other body fluids and is responsible for millions of infections worldwide. Mostly targeting CD4^+^ T cells, HIV quickly intrudes these cells (within a few days) and inserts into their genome, which induces the transition of CD4^+^ T cells into a “quiescent” state, leading to generalized immunosuppression [24]. If it remains untreated, this infection results in the death of the patient after several years. Antiretroviral therapy (ART) has undeniably revolutionized the treatment of HIV and is currently the most widespread treatment for controlling the disease and for limiting its transmission. Nevertheless, this therapy, although revolutionary, has limitations, including toxicity due to life-long administration, as well as resistance in some patients [14]. Moreover, ART is not a functional cure because the virus persists in long-lived CD4^+^ T cells, even when the patient is under treatment [15].

In HIV infection, T cells (and more specifically, CD8^+^ cytotoxic T cells) play a significant role in enhancing the immune response. This effect is evidenced not only by the association between various HLA class I alleles (expressed by most nucleated cells of the organism) and the control of HIV but also by the formation of HIV-specific CD8^+^ T cells with memory potential that decrease viremia early in infection [25]. Consistently, several T-cell immunotherapeutic approaches, specifically vaccines, cytokines, immune checkpoint blocking agents and CAR-T cells, are being studied for the treatment of HIV infection, as recently reviewed [25].

Consistent with the important role of T cells in HIV infection, different antibodies engaging T cells through CD3 and infected cells have been developed with HIV-specific epitopes derived from broadly neutralizing and nonneutralizing antibodies.

In 2015, Sung et al. [24] and Sloan et al. [26] developed a series of anti-HIV × anti-CD3 dual affinity retargeting (DART) proteins that engage cytolytic effector T cells in HIV-1-infected cells, notably through gp120 or gp41 expressed by the HIV envelope. Both groups reported encouraging in vitro results, as they could redirect cytotoxic CD8^+^ T cells toward HIV-infected cells and reactivate ex vivo HIV protein expression [6, 15]. The combination of HIVxCD3 DART molecules further leveraged the host immune system for the treatment of HIV-1 infection but still required appropriate reactivation of the latent reservoir by latency reversing agents (LRAs) to be fully effective at curing HIV [27]. Three years later, Brozy et al. [28] also conceived an anti-gp120 × anti-CD3 molecule (in a BiTE format) and were able to redirect T cells to gp120-positive HIV cells in vitro, as well as to inhibit viral replication.

Subsequently, the DART molecule MGD014, which targets CD3 and the C1/C2 regions of the gp120 subunit, was registered for evaluation in a phase I clinical trial in 2022 (NCT03570918). Moreover, a second phase I study comparing the use of MGD020 (DART anti-CD3 x anti-gp41 subunit) alone or in combination with MGD014 in patients with HIV on ART was launched in March 2022, and participants are currently being recruited (NCT05261191) to obtain ultimate proof of reservoir reduction in vivo.

Based on the trispecific anti-CD3 × anti-CD28 × anti-CD38 engager (SAR442257) developed in the cancer field, Promsote et al. recently developed an anti-CD3 × anti-CD28 × N6 agent that targets the CD4 binding site of the HIV envelope while recruiting and activating T cells [29]. This construct represents the first trispecific T-cell engager reported for HIV treatment and produces enhanced reactivation and elimination of latently infected cells from ART-suppressed HIV^+^ donors. In macaques, the molecule was well tolerated and led to robust activation of CD4^+^ and CD8^+^ T cells in both peripheral and secondary lymphoid tissues [29].

In addition to HIV, interest in hepatitis B virus (HBV) has increased in the field of T-cell engagers. HBV is a virus often acquired at birth through vertical transmission (but can also be transmitted sexually and parenterally through blood and blood products) and is associated with severe chronic complications, including cirrhosis and liver cancer [30]. Although efficient vaccines exist, HBV is still responsible for approximately 820,000 deaths every year due to a lack of diagnosis or access to prophylactic therapy with antivirals [22]. Because of an intracellular viral replication intermediate named covalently closed circular (ccc) DNA, current antiviral therapies rarely achieve a cure. Upon infection, cccDNA is produced as a plasmid-like episome in the host cell nucleus and serves as a template for all viral RNAs of new virions. Therefore, novel anti-HBV treatments are still necessary, and the use of a multispecific T-cell engager might be a reasonable approach to counteract the limitations observed with antiviral nucleos(t)ide therapy, IFN-α-based approaches or CAR-T cells [30].

The first mention of a T-cell engager for the treatment of HBV was reported by Liao et al. in 1996, revealing the early interest in such a molecule in this context [31]. Then, in 2017, Kruse and collaborators reported the administration of plasmids encoding the bispecific antibody CD3 × HBsAg (HBV-specific antigen) into the liver of HBV-infected mice, which significantly decreased HBV-driven reporter gene expression and increased host IgG antibody production against HBsAg [32]. Although preliminary, this study revealed the promising future of gene therapy with bispecific T-cell engagers for treating HBV infection. In 2021, two bispecific antibodies targeting HBV envelope proteins (HBVEnv) were further developed: one with an anti-human CD3 antibody to recruit T cells and the other with an anti-CD28 antibody to provide the costimulatory signal necessary for T-cell activity against the virus [30]. The authors reported significant in vitro results, as they not only showed a redirection of T cells to infected cells but also T-cell activation (measured by granzyme B and IFN-γ), resulting in important cytotoxicity toward HBVEnv-expressing tumors in C57BL/6 mice. A tri-specific antibody combining anti-HBVEnv × anti-CD3 × anti-CD28 molecules was subsequently generated by the same authors but without an enhanced effect compared to the combination of the two bispecific antibodies [22]. This result could be explained by the steric hindrance that impedes the engagement between the three antigens [22]. While more studies are still needed to improve the structure and functionality of these constructs, T-cell engagers are clearly of interest and are promising for HBV therapy.

Other viral diseases, even coronavirus disease 2019 (COVID-19), which is a disease caused by the virus SARS-CoV-2 and is responsible for a worldwide pandemic causing over 6 million deaths in the last few years, have been the focus of research in the T-cell engager field. As early as 2022, Dogan et al. designed an anti-CD3 x anti-Spike agent using the extracellular domain of angiotensin-converting enzyme 2 (ACE2), the cellular receptor for SARS-CoV-2, in an attempt to overcome the practical limitations of CAR-T-cell therapy [33]. Increased T-cell activation (measured by CD25 expression) and target cell killing in the presence of ACE2-expressing cells and the BiTE were shown in vitro. Interestingly, the authors showed that the BiTE acts as a decoy receptor for SARS-CoV-2, leading to neutralization of the virus and therefore preventing its entry into the host cell independent of the variants of the Spike protein [33]. One year later, Li et al. reported the development of another SARS-CoV-2 spike-targeting bispecific T-cell engager (S-BiTE) and tested its efficacy both in vitro and in vivo [34]. Aside from its ability to stimulate T-cell cytotoxicity against cells infected with both the original variant and the Delta variant, the S-BiTE administered to humanized mice infected with SARS-CoV-2 was able to decrease the viral load, an encouraging result for the use of BiTEs for the treatment of COVID-19.

Finally, treatment of human cytomegalovirus (HCMV) could also benefit from multispecific T-cell engagers. While HCMV primoinfection is usually infrequent in healthy individuals, this viral infection becomes particularly problematic when the latent virus is reactivated in immunocompromised patients, such as in recipients of allogeneic hematopoietic stem cell transplantation (HCST). In this context, massive viral replication and dissemination can occur, leading to complications such as increased risks of graft-versus-host disease, bacterial and fungal infections, and ultimately life-threatening consequences if not treated properly [35, 36]. Treatment options include antiviral medications (such as ganciclovir), which are associated with toxicity and drug resistance, and adoptive immunotherapy with HCMV-specific T cells, which is associated with practical difficulties and interindividual variability in efficacy [35, 36]. To overcome these limitations, Two teams reported the development of BiTE against HCMV-infected cells in 2018 to overcome these limitations [35, 36]. Meng et al. and Brey et al. both developed an anti-CD3 x anti-glycoprotein B (gB) BiTE and demonstrated similar outcomes, including i) the redirection of T cells toward HCMV-infected cells, ii) the stimulation of T-cell activation measured by the production of TNF-α and IFN-γ in the culture supernatant, and iii) the viral inhibition of HCMV in vitro. Nonetheless, no lysis of the infected cells was observed in the presence of the bispecific construct, underlining the difficulty of tackling this infection and the necessity for further investigations.

Scientific and clinical advances in T-cell immunotherapy for cancer treatment are well ahead of those for viral diseases. This principle is even the case for HIV, which is the most advanced among the viral diseases mentioned in this review. While different T-cell engagers have already received approval from regulatory authorities against cancer, these molecules are still in preclinical stages for viral diseases. The translation from cancer to viral infection, such as HIV or HBV, poses additional challenges, including the persistence of viral reservoirs, high viral diversity, immune evasion, chronic immune activation and immune exhaustion, and these challenges are at the root of the current lack of clinical benefits of treatment with T-cell immunotherapies for an HIV or HBV cure [11, 37, 38]. In this regard, T-cell immunotherapies, and more specifically T-cell engagers, still need to be carefully improved and/or used in combination with antiretroviral therapy or potent LRAs to reactivate and kill the HIV reservoir to achieve a functional HIV cure (as defined by “durable virologic control”). Current combined antiretroviral therapy controls active HIV replication and decreases the plasma viral load to an undetectable level. However, treatment interruption ultimately leads to viral rebound, which makes antiretroviral therapy noncurative [39] but helps to decrease the number of viral reservoirs integrated or not integrated into genomic DNA and further containment by the immune response. In the context of functional HBV infection, the persistence of reservoirs of HBV replication and antigen production (HBV DNA) causes a high burden of viral antigens, resulting in T-cell exhaustion and dysfunction, as well as a chronic hepatitis B-induced alteration of immune responses. A number of novel and promising approaches targeting high viral DNA or antigen burdens and restoring an effective immune response are being explored. Similar to those for HIV, combination regimens with direct-acting antiviral drugs or immunotherapy are likely to be required because of the many ways in which the hepatitis B virus can evade the immune system. Therefore, restoring a functional adaptive immune response toward HBV treatment remains highly challenging without a sterilizing cure allowing the eradication of HBV DNA and the subsequent loss of HBV surface antigen [37, 38].

T-cell engagers, whether they are used in the context of cancer or viral diseases, are also associated with several limitations. First, the safety profile of these molecules is a matter of concern, as several adverse events (AEs), including cytokine release syndrome (CRS) and neurotoxicity, have been detected in patients who receive T-cell-engaging immunotherapies [18]. In the worst cases, these AEs can lead to multiorgan failure and ultimately to patient death. In addition, the pharmacokinetic properties of T-cell engagers can be problematic due to the limited biodistribution of the molecules in the targeted tissue, as well as their limited half-life [7]. However, these properties could be improved by modulating the structure of the engagers by adding, for instance, an Fc domain, albumin or polyethylene glycol to the construct [7]. T-cell-engaging immunotherapies are also associated with the onset of various resistance mechanisms, such as the appearance of mutations at the targeted site, which can lead to treatment failure. Finally, particularly in cancer, the specific tumor microenvironment (TME) can hinder the infiltration of T cells and produce exhaustion molecules that impair the efficacy of T-cell engagers [6, 7].

Despite these limitations, T-cell engagers still have a bright future. The development of new target sites of conditionally active T-cell engagers (delivered as inactive prodrugs and activated only at the site of interest) or the development of various T-cell-enhancing methods represent potential optimizations of the current therapy that could increase the efficiency of T-cell-engaging immunotherapies against cancer and viral infections [13].

## Multispecific engagers binding NK cells in cancer and infectious diseases

Natural killer (NK) cells represent the third type of lymphocyte, in addition to T and B cells. Originating from common lymphoid progenitors, these cells take their name from their natural ability to kill cancer cells in vitro [40]. In contrast to T and B cells, the recognition and activation of NK cells toward their target is based on a balance between activating and inhibitory signals. Indeed, NK cells possess an array of activating and inhibitory receptors that are able to perceive whether a target cell is missing its MHC-I (“missing-self hypothesis”) or expressing abnormal stress molecules, both of which result from the abnormal behavior of the cell (malignant or virus-infected) [40, 41]. Upon activation, NK cells act in different ways to kill their target. One of their main cytotoxic effects is the release of perforin and granzyme-containing granules, which are able to form pores and trigger apoptosis in targeted cells. NK cells are also potent producers of chemokines and cytotoxic cytokines (such as TNF-α and IFN-γ) and are subsequently able to trigger antibody-dependent cellular cytotoxicity (ADCC) due to the presence of FcγRIIIa (CD16a) on their surface [41, 42].

Compared with T-cell immunotherapy, NK-cell immunotherapy has many advantages, mainly because of its safer toxicity profile. Allogeneic transfusion of CAR-NK cells in patients does not trigger graft-versus-host disease (GvHD), nor does it stimulate cytokine release syndrome (CRS), two serious side effects often observed in T-cell immunotherapy, which can even be fatal, as recently reported [43, 44]. Moreover, NK cells do not require a preimmunization process (making them a good “off-the-shelf” approach) and can be obtained from a variety of sources [45].

**Fig. 4:**
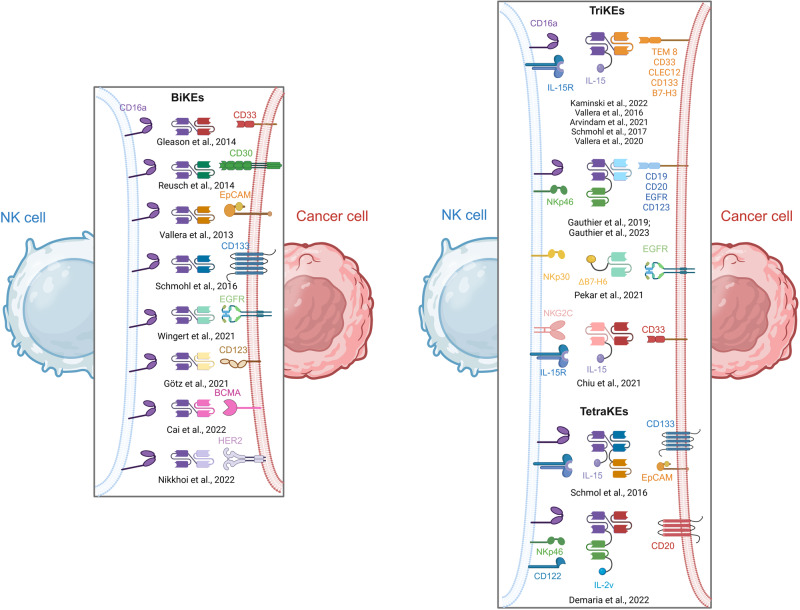
Overview of NK-cell engagers (NKCEs) in cancer. NKCEs are generally composed of single-chain variable fragments (scFvs) or specific ligands directed against i) NK cell targets such as CD16a (purple), IL-15R (blue and purple), NKp46 (green), NKp30 (yellow), NKG2C (pink) or CD122 (dark blue), and ii) tumor-associated antigens such as CD33 (red), CD30 (green), EpCAM (yellow and orange), CD133 (dark blue), EGFR (light green), BCMA (pink), HER2 (light purple) or CD123 (light yellow). BiKE bispecific killer engager, TriKE trispecific killer engager, TetraKE tetraspecific killer engager, EGFR epidermal growth factor receptor, BCMA B-cell maturation antigen, HER2 receptor protein tyrosine kinase erbB-2, TEM8 tumor endothelial marker 8, CLEC12 C-type lectin domain family 12 member A, IL-15R IL-15 receptor, IL-2v IL-2 variant. Created with Biorender

Given all the advantages of these innate cells, which produce a fast and robust response that could generate further protective adaptive immunity, many efforts are currently deployed to stimulate the function of NK cells in various disease models, notably through NK cell engagers (NKCE). Their inhibitory and activating receptors, although not always specific to NK cells, represent attractive targets for this therapy, and many NKCEs are currently being developed and are summarized in Fig. 4 [40].

### Bispecific killer engagers (BiKEs)

A popular and attractive target for NK cells is CD16a, the receptor that recognizes the Fc fragment of IgG antibodies and confers to NK cells their ability to perform ADCC [43]. Among the bispecific killer engagers (BiKEs) developed for cancer treatment, Gleason et al. reported the development of anti-CD16a × anti-CD33 for the treatment of myelodysplastic syndromes (MDS) [46], and Reusch et al. developed a tetravalent bispecific anti-CD16a × anti-CD30 compound for the treatment of Hodgkin Reed–Sternberg (HRS) lymphoma cells [47]. Interestingly, because of its bivalency toward CD16a and CD30, this BiKE could bind longer on the surface of NK cells and be more potent and effective than anti-CD30 antibodies while maintaining its specificity against CD30^+^ targets [47]. BiKEs targeting CD16a were also directed against various TAAs, such as EpCAM (for EpCAM-expressing carcinoma) [48], CD133 (colorectal cancer-associated cancer stem cells) [49], EGFR (EGFR^+^ solid tumors) [50], CD123 (acute myeloid leukemia) [51], BCMA (multiple myeloma) [52] and, recently, HER2 (HER2^+^ tumors) [53]. The evaluation of such constructs in vitro encompasses various outcome measures, such as specific binding to targeted cells, NK cell activation, degranulation, proinflammatory cytokine production, and enhanced cytotoxicity against tumor cells, which could be observed for all these molecules.

The tolerance and safety profiles of certain BiKEs, which exhibit exceptional in vitro results, were further evaluated in nonhuman primates (NHPs), an indispensable step preceding clinical studies. The constructs that could safely be administered were further included in clinical studies, as discussed in other reviews [5, 43]. As such, AFM13 (anti-CD16a × anti-CD30, phase II), AFM24 (anti-CD16a × anti-EGFR, phase I), AFM26 (anti-CD16a × anti-BCMA, phase I) and AFM28 (anti-CD16a × anti-CD123, pre-IND) are currently being evaluated for the treatment of lymphoma, advanced solid cancers and acute myelogenous leukemia (AML), respectively.

In addition to their role in antitumor immunity, NK cells also have important functions in the fight against virus-infected cells [54]. Accordingly, several NK-based immunotherapies, such as antibodies (which either block inhibitory or boost activating receptors) and CAR-NK cells that retarget NK cells to HIV-infected cells, are being tested in HIV infection settings [54]. Among other approaches, our team also recently reported the development of a molecule able to boost NK cells and stimulate their cytotoxic action against HIV-infected cells by binding to the inhibitory receptors NKG2A or KIR and by multimerization of IL-15 [55].

**Fig. 5:**
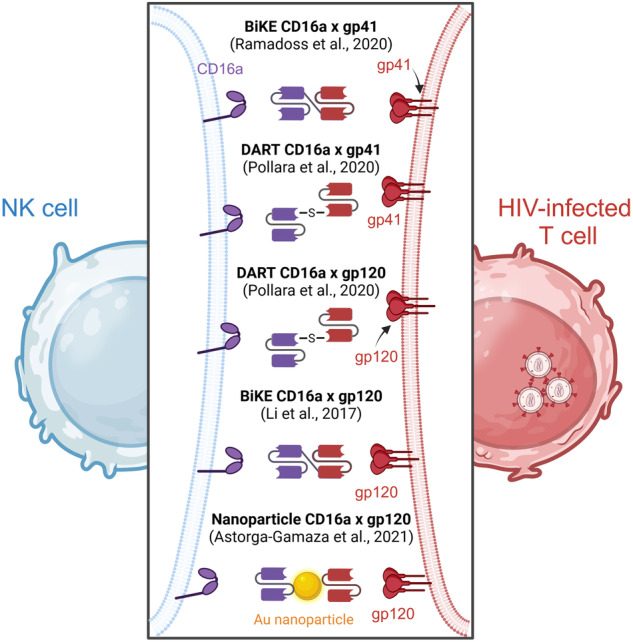
NK cell engagers targeting HIV-infected T cells. Bispecific killer engagers (BiKEs), dual affinity retargeting (DART) proteins or gold (Au) nanoparticles are targeted against NK cells via CD16a and against HIV-infected T cells via anti-gp41/anti-gp120 binding moieties. Created with Biorender

A few BiKEs have been developed for HIV treatment (Fig. 5), including a construct presenting moieties that bind to CD16a and to gp41, an antigen expressed on the surface of HIV-infected cells that forms a specific structure called a “stump” [56]. In this study, Ramadoss et al. showed that NK cells incubated with the BiKE exhibit increased degranulation (as evidenced by increased CD107 expression) and cytotoxicity toward HIV-infected cells, as measured by calcein release [56]. The same year, another team reported the design of two anti-HIV × anti-CD16 (against gp41 or against gp120) DARTs able to retarget neonatal NK cells (obtained from human umbilical cord blood) to kill autologous HIV-infected T cells as a model of the mother-to-child transmission of the virus [57]. Other studies reported the characterization of BiKEs in HIV infections, such as an anti-CD16a × one-domain soluble CD4 antibody by Li et al. [58] or, more recently, bispecific gold (Au) nanoparticles coupling an anti-gp120 antibody and an anti-CD16a antibody [59], indicating the increasing interest in NK cell multispecific engagers for the treatment of HIV infection.

### Trispecific killer engagers (TriKEs)

Similar to what has been performed for T cells, BiKEs have also been optimized into tri-specific killer engagers (TriKEs). For the third moiety of TriKE, IL-15 is an attractive candidate, as this cytokine is highly involved in NK cell development and function [60]. Therefore, many teams have improved their bispecific construct by adding IL-15, such as the anti-CD16a × IL15 × anti-TEM8 developed by Kaminski and collaborators [61]. As TEM8 is expressed on the surface of tumors, tumor stromal cells, and endothelial cells and fibroblasts of the tumor microenvironment, this approach aims to overcome the physical and biological barriers of the tumor stroma [61]. Other constructs include anti-CD16a × IL15 × anti-CD33 [60] and anti-CD16a × IL15 × anti-CLEC12 [62], both of which were developed for the treatment of AML, anti-CD16a × IL15 × anti-CD133 for various carcinomas [63], and anti-CD16a x IL15 x anti-B7-H3, an antigen expressed on the surface of different tumors but studied in this report in the frame of ovarian, prostate and lung cancers [64]. While all these constructs showed good preclinical outcomes (specific activation and cytotoxicity of NK cells toward their targets in various in vitro models and in mice), the construct GTB-3550 (CD16 × IL15 × CD33) showed particularly promising results and was evaluated in a first-in-human phase I clinical trial for AML treatment. This trial revealed a safe and well-tolerated response at the doses given to the participants (NCT03214666). However, the development of this construct is currently halted, as the company is developing an alternative format for their therapy using camelid nanobody technology against the same target [65].

With a new approach, Gauthier and collaborators added a second scFv directed against another NK-activating receptor, NKp46. Compared to CD16a or NKG2D, this receptor is almost exclusively expressed on NK cells, increasing the specificity of their construct. Moreover, previous reports described the necessity of the coengagement of different receptors for the complete activation of NK cells, thus supporting their strategy to target CD16a and NKp46 simultaneously [66]. In a first paper in 2019, they developed anti-CD16a × anti-NKp46 × anti-CD19/CD20/EGFR constructs (the three formats were produced, compared and tested) and showed NK cell activation and specific antitumor effects in vitro and in vivo (on tumor-injected SCID mice) [66]. Recently, the same team published the development of another construct, anti-CD16a × anti-NKp46 × anti-CD123, which showed elegantly favorable in vivo results, including the control of tumor growth in mice and, importantly, a safe toxicity profile in NHPs (cynomolgus monkeys), as evidenced by the low release of the cytokines IL-6 and IL-10 and the absence of associated clinical signs [67]. Overall, this study supported the clinical development of anti-CD16a x anti-NKp46 × anti-CD123 (IPH6101/SAR443579) for the treatment of AML, which is currently part of a phase I/II clinical trial to evaluate the safety, pharmacokinetic and pharmacodynamic properties, and anticancer activity of this molecule against different blood tumors (NCT05086315).

In addition to CD16a and NKp46, other NK-activating receptors can be used as targets for NK-cell engagers. In another approach, rather than using an scFv against NKp30, the authors modified its natural ligand B7-H6 to enhance NKp30 binding [68]. In addition, NKG2C was also explored as a therapeutic target in preclinical AML studies using the construct anti-NKG2C × IL15 × anti-CD33 [59]. This approach was chosen due to concerns about the lack of specificity of CD16a, which is also found on the surface of neutrophils and may result in off-target effects. In contrast, NKG2C is an NK-activating receptor that can trigger potent NK cell activation but is almost exclusively expressed on a specific subtype of resting NK cells. Because these cells represent only a small proportion of primary NK cells, the authors propose a dual approach composed of TriKE combined with the administration of NKG2C^+^ induced pluripotent stem cell (iPSC)-derived NK (iNK) cells. In this setting, the authors report enhanced degranulation, IFN-γ production and cytotoxicity of the iNK treated with the TriKE toward AML targets in vitro. This effect was observed only on iNKs transfected with NKG2C, highlighting the necessity of high NKG2C expression for the action of the TriKE [69].

### Tetraspecific killer engagers (TetraKEs)

Finally, Schmol et al. added a fourth moiety to their construct and designed a tetraspecific NK-cell engager targeting CD16a, two tumor antigens (EpCAM and CD133) to target both carcinoma cells (through EpCAM) and cancer stem cells (through CD133), and IL15 to enhance NK cell activation [70]. Another tetraspecific killer engager called IPH6501 composed of anti-NKp46 × anti-CD16a × anti-CD20 × anti-CD122 (the latter being the β chain of the IL-2R) was further designed [71, 72]. The authors reported increased cytotoxicity of NK cells toward CD20^low^ cells in vitro, potent antitumor activity of their construct in xenograft mouse models engrafted with the Raji cell line (human B-cell lymphoma), and a safe toxicity profile in NHPs [71, 72]. These results suggests that tetraspecific constructs might represent the next wave of NKCEs, given their apparent superiority to BiKEs and TriKEs.

The growing interest in NK cells has greatly motivated the development of NK-cell engagers, particularly in cancer immunotherapy. Due to their favorable toxicity profile, as well as their targeted action demonstrated in numerous preclinical studies, several bi and tri-specific NKCEs have even entered clinical trials, highlighting the great potential of these constructs in cancer immunotherapy.

Although their effectiveness in human studies remains to be proven, combining their usage with other strategies (such as cytokines or CAR-NK cells) might be necessary to attain a satisfactory level of efficacy, given that these cells are present in lower quantities than T cells. Additionally, the majority of studies have focused on liquid cancers, emphasizing the challenges associated with addressing solid tumors, such as antigen accessibility and a hostile tumor microenvironment. Nonetheless, NKCEs hold great potential for the future of cancer immunotherapy, and the results from various ongoing clinical trials should provide us with more insights into its use as a cancer treatment.

To the best of our knowledge, only a few BiKEs have been reported in the context of HIV infection. Despite the key role of NK cells in antiviral immunity, the development of NKCEs in this context still seems to be in its very early stages, and more studies are needed to assess whether this approach is relevant for the treatment of HIV, particularly when viral persistence is mainly restricted to latent memory CD4^+^ T cells. Moreover, while the antiviral action of NK cells is not restricted to HIV-infected cells, a surprising absence of studies reporting the development of NK-cell engagers for other infectious diseases has been noted. This lack of research leaves the door open to many possibilities for the development of NKCEs for the treatment of other infectious diseases, which could hopefully overcome these limitations or could even work synergistically with T-cell immunotherapy.

## Multispecific engagers binding myeloid cells in cancer and infectious diseases

The innate immune system (notably macrophages, neutrophils and dendritic cells) unquestionably possesses potential for therapy. It represents the first line of action against pathogens and tumors, as it acts through germline-encoded pattern recognition receptors (PRRs), allowing them to fight the intruder quickly, although not specifically [73]. Moreover, activation of the innate immune system is crucial for proper activation of the adaptive immune system, notably through the presentation of antigens by antigen-presenting cells (APCs) to naïve T and B lymphocytes [73].

### Macrophages and neutrophils

Macrophages, a subset of tissue cells derived from circulating monocytes, can perform phagocytosis, i.e., engulf undesirable material such as debris or pathogens through the formation of pseudopods. This process is extremely important both physiologically (to remove debris and ensure tissue homeostasis) and pathologically (to kill bacteria or malignant cells, for example) [73].

In cancer, the role of macrophages is not always clear, as macrophages sometimes have a beneficial effect (M1 macrophages showing a proinflammatory profile that contributes to a hostile environment for the tumor) but are able to promote tumor growth in other contexts (M2 macrophages possessing anti-inflammatory properties) [73–75]. Nonetheless, macrophages are involved in cancer cell removal due to their expression of all classes of Fcγ receptors (unlike NK cells that express only FcγRIIIa), allowing them to perform antibody-dependent phagocytosis (ADCP) [74], as well as the expression of the IgA receptor FcαRI (CD89), which has various functions, including ADCC, phagocytosis and mediation of inflammation and cytokine release [76].

Neutrophils are the first cells to be recruited to a site of infection. Like macrophages, they have a controversial role in tumor responses. These cells were initially classified as “N1 neutrophils” (with a proinflammatory and antitumor phenotype) or “N2 neutrophils” (with immunosuppressive and protumorigenic properties) [75], but this simple classification has largely evolved, with more than 19 neutrophil subtypes identified [77]. Neutrophils can kill tumor cells through various mechanisms, including phagocytosis, degranulation (production and release of various granules containing effectors such as hydrolytic enzymes, defensins and metalloproteases), neutrophil extracellular traps (NETs) (release of neutrophil-nuclear components and cytotoxic factors to trap pathogens), and trogocytosis (internalization of parts of the tumor cell membrane, inducing cell death via necrosis) [75]. This wide variety of cytotoxic activities of neutrophils toward cancer cells can then be used and enhanced by bispecific antibodies and neutrophil engagers.

Since neutrophils are also characterized by the expression of different FcγR and FcαRI receptors, the finding that macrophage and neutrophil engagers are approached through similar approaches is not surprising. Many bispecific engagers targeting these cell types have been studied in preclinical and clinical studies, which have been brilliantly reviewed elsewhere [5, 75] and are summarized in Table 1.

**Table 1:** Summary of myeloid cell engagers described in cancer and HIV

Targeted immune cell	Targeted antigens	Disease	Main results in preclinical studies	Clinical trial (highest phase)	Refs.
Macrophages and neutrophils (FcγRI)	Anti-CD30 × anti-FcγRI (H22xKi4)	Lymphoma	In vitro:	Yes, Phase I (discontinued)	[109, 110]
			• H22xKi4 binds to CD30^+^ cells		
			• H22xKi4 potently mediates ADCC with CD30^+^ tumor cells and human monocytes		
			• H22xKi4 enhances monocyte-derived macrophages-mediated phagocytosis		
	Anti-HER2/neu × anti-FcγRI (H22x520C9/MDX-210)	HER2^+^ breast, ovarian or prostate cancers	In vitro:	Yes, Phase II (discontinued)	[111–113]
			• H22x520C9/MDX-210 mediates ADCP and ADCC in the presence of monocyte-derived macrophages (MDM) against HER2^+^ target cells at similar levels than monoclonal antibody against anti-HER2/neu		
	Humanized anti-HER2 × anti-FcγRI (MDX-H210)	HER2^+^ breast, ovarian or prostate cancers	In vitro:	Yes, Phase II (discontinued)	[111, 112, 114]
			• MDX-H210 mediates ADCP to a similar level than MDX-210		
	Anti-EGFR × anti-FcγRI (MDX-447)	EGFR^+^ tumors	In vitro:	Yes, Phase II (discontinued)	[115, 116]
			• MDX-447 binds to EGFR^+^ and FcγRI^+^ cells		
			• MDX-447 mediates ADCC and lysis of EGFR-overexpressing cell lines		
	Anti-EpCAM × anti-FcγRI (HEA125x197)	Ovarian carcinoma and other EpCAM^+^ carcinomas	In vitro:	No	[117]
			• HEA125x197 binds to EpCAM^+^ and FcγRI^+^ cells		
			• HEA125x197 induces potent cytotoxic activity towards allogeneic and autologous ovarian carcinoma cells in the presence of stimulated CD64^+^ polymorphonuclear neutrophils (PMN)		
	Anti-gp41 × anti-FcγRI (MDX-240)	HIV	In vitro:	Yes, Phase II (discontinued)	[87]
			• MDX-240 mediates viral inhibition infection of PBMCs and human macrophages		
			• MDX-240 reverses ongoing in vitro HIV-1 human macrophage infection		
Macrophages and neutrophils (SIRPα)	Anti-SIRPα × anti-CD70	Non-Hodgkin lymphoma, multiple myeloma, renal cell carcinoma, and glioblastoma	In vitro:	No	[78]
			• The construct enhances macrophage phagocytosis of renal carcinoma cells, an effect not observed with the combination of monoclonal antibodies anti-SIRPα and anti-CD70		
			In vivo:		
			• The construct inhibits Burkitt’s lymphoma cell growth in SRG mice, but not better than the combination of monoclonal antibodies		
Macrophages and neutrophils (FcαRI)	Anti-CD30 × anti-FcαRI (A77xKi4)	Lymphoma	In vitro:	No	[109]
			• A77xKi4 binds to CD30^+^ cells		
			• A77xKi4 potently mediates ADCC of CD30^+^ tumor cells by human monocytes		
			• A77xKi4 partially mediates ADCC of freshly prepared PMN leukocytes against CD30^+^ cells		
			• A77xKi4 enhances monocyte-derived macrophages-mediated phagocytosis		
	Anti-HER-2/neu × anti-FcαRI (A77x520C9)	HER2^+^ carcinomas	In vitro:	No	[118, 119]
			• A77x520C9 induces phagocytosis of breast cancer cell lines by human macrophages, at similar rate than H22x520C9 (anti-HER2/neu × anti-FcγRI)		
			• Macrophages treated with GM-CSF, but not INF-γ, induce more efficient phagocytosis of breast cancer cells by macrophages in presence of A77x520C9		
			• A77x520C9 induces breast cancer cell lines autophagy (and not apoptosis) when incubated with human PMN		
	Anti-CD20 × anti-FcαRI	B cell malignancies and lung cancer	In vitro	No	[76]
			• The construct binds to CD20^+^ cells (Raji cells) and FcαRI^+^ cells (PMN)		
			• The construct mediates ADCC of Raji cells via human PMN		
			In vivo		
			• The construct enhances the regression of Raji cells tumors in NOD/SCID mice in the presence of PMN		
			• Tumor cell killing of Lewis lung cancer (LLC) cells transfected with human CD20 (LLC-hCD20) in FcαRI Tg mice is enhanced in the presence of the bispecific construct		
			• Tumor associated macrophages (TAM) isolated from LLC-hCD20 mice mediate ADCP of Raji cells in the presence of the bispecific construct		
	Anti-HER-2 × IgGA	HER2^+^ carcinomas	In vitro	No	[82]
			• The construct binds to FcαRI, FcγRI and FcγRIIa due to engineered “cross-isotype” antibody IgGA		
			• The construct mediates HER2^+^ cancer cells killing by ADCC and ADCP, by both macrophages and neutrophils		
			• Complement-dependent cytotoxicity (CDC) is improved in the presence of the cross-isotype construct compared to IgG1 or IgA antibodies		
	Anti-HER-2 × IgG1/IgA2	HER2^+^ carcinomas	In vitro	No	[83]
			• The construct stimulates ADCC activity of NK cells and freshly isolated PMN cells against HER2^+^ cells		
			• ADCP activity by macrophages against SK-BR3 and MDA-MB-453 cells is enhanced in the presence of the construct		
			• The construct increases the recruitment and cytotoxic functions of PMN against HER2^+^ cell lines in comparison to IgG1 or IgA2		
			In vivo		
			• The construct shows improved pharmacokinetic properties in BALB/c mice compared to parental IgA2 (better serum persistence)		
	Anti-CD20 × IgGA	B cell malignancies	Ex vivo	No	[84]
			• Tumor cell killing of Raji cells by both human myeloid effector cells and Tg is enhanced in thepresence of the IgGA construct compared to CD20-IgG or CD20-IgA		
			In vivo		
			• FcαRI Tg mice treated with the IgGA construct show enhanced tumor cell killing of Lewis lung cancer (LLC) cells transfected with human CD20 (LLC-hCD20), compared to CD20-IgG or CD20-IgA		
	Anti-EGFR × anti-FcαRI × anti-FcγRI (TrisomAb)	Colorectal cancer	In vitro	No	[85]
			• anti-EGFR TrisomAb induces FcγRI mediated ADCC (by NK cells) and ADCP (by macrophages) of EGFR^+^ cells		
			• anti-EGFR TrisomAb induces FcαRI mediated cytotoxicity of EGFR^+^ cells by neutrophils		
			• Colorectal cancer patients-derived neutrophils effectively eliminates tumor cells in the presence of anti-EGFR TrisomAb		
	Anti-gp75 × anti-FcαRI × anti-FcγRI (TrisomAb)	Melanoma	In vitro		
			• anti-gp75 TrisomAb induces FcγRI mediated ADCC (by NK cells) and ADCP (by macrophages) of gp75^+^ cells		
			• anti-gp75 TrisomAb induces FcαRI mediated cytotoxicity of gp75^+^ cells by neutrophils		
			In vivo		
			• Tumor outgrowth in FcαRI transgenic C57BL/6 mice injected subcutaneously with gp75^+^ cells is reduced in the presence of TrisomAb, by macrophages, NK cells and neutrophils		
	Anti-gp41 × anti-FcαRI	HIV	In vitro	No	[91]
			• The bispecific-antibody-mediated destruction of HIV and HIV-infected cells by ADCVI (antibody-dependent cell-mediated virus inhibition) is induced in the presence of neutrophils		
Dendritic cells	Anti-CD40 × anti-EpCAM (Neo-X-Prime/ATOR-4066)	EpCAM^+^ cancer	In vitro	No	[97]
			• Binding on antigen-presenting cells (APC) and EpCAM^+^ cancer cells resulting in activation of APC		
			• Stimulation of delivery of necrotic tumor debris to APCs, which was not observed with monoclonal antibody against EpCAM		
			In vivo		
			• hCD40tg mice bearing MB49-EpCAM tumors and treated with anti-CD40 × anti-EpCAM show EpCAM-dependant anti-tumor effects as compared to CD40 mAb or isotype × EpCAM		
			• Administration of anti-CD40 × anti-EpCAM stimulates immunological memory in tumor bearing mice and prevents the growth of new MB49-EpCAM tumors		
			• No systemic inflammation is associated with the administration of anti-CD40 × anti-EpCAM in mice and non-human primates		
	Anti-CD40 × anti-CEA (Neo-X-Prime/ATOR-4066)	CEA^+^ cancer	In vitro		
			• Anti-CEA ATOR-4066 binds to antigen-presenting cells (APCs) and CEA^+^ cancer cells resulting in activation of APC		
			• Anti-CEA ATOR-4066 stimulates the delivery of necrotic tumor debris to APCs, which was not observed with monoclonal antibody against CEA		
			In vivo		
			• hCD40tg mice injected with CEA-transfected MC38 cells and administered with anti-CEA ATOR-4066 showed significant anti-tumor effects		
	Anti-CD40 × anti-CEA	CEA^+^ cancer	In vitro/Ex vivo	No	[96]
			• Anti-CD40 × anti-CEA binds specifically to their targets and induces CEA-dependent CD40 agonism of splenic DC isolated from huCD40tg mice, resulting in enhanced T cell cross-priming		
			• Anti-CD40 × anti-CEA promotes the delivery of CEA^+^ beads and CEA^+^ tumor-derived extracellular vehicles (EVs) to DC, facilitating the presentation of tumor antigen and ultimately the tumor-specific T cell priming		
	Anti-CD40 × anti-MSLN (ABBV-428)	MSLN^+^ cancer	In vitro	Yes, Phase I (NCT02955251)	[100, 120]
			• ABBV-428 induces CD40-dependent APCs activation and proliferation, only when co-cultured with MSLN^+^ cells		
			• ABBV-428 induces MSLN-dependent T cell activation		
			• The efficacy of ABBV-428 is dependent on the amount of MSLN expression, that should be above a specific threshold		
			In vivo		
			• NSG mice inoculated with MSLN^+^ tumor cells and treated with ABBV-428 show tumor regression in a specific manner		
	Anti-LAG3 × PDL-1 (ABL501)	Progressive, locally advanced (unresectable) or metastatic solid tumors	In vitro	Yes, Phase I (NCT05101109)	[99]
			• ABL501 simultaneously blocks LAG-3 and PD-L1 and efficiently activates CD4^+^ and CD8^+^ T cells		
			• ABL501 compensates T_reg_-cell suppressive functions towards effector T cell, with better efficacy than the monoclonal antibodies		
			• CD8^+^ T cell activation by ABL501 is explained by enhanced DC maturation and increased conjugation between T cells and tumor cells		
			In vivo		
			• Humanized NSG mice injected with A375-PD-L1 tumors, adoptively transferred with 1G4 TCR-T cells and administered with ABL501 showed significant tumor regression as compared to anti-PD-L1 treatment, as well as increased tumor-infiltrating lymphocytes (TILs) rate and activation		
			• ABL501 presents a good safety profile in mice and in cynomolgus monkeys		
	Anti-CD40 × anti-FAP (MP0317)	Advanced solid tumors	In vitro	Yes, Phase I (NCT05098405)	[101]
			• MP0317 specifically activates APC in presence of FAP^+^ cells		
			In vivo		
			• Mice with FAP^+^ tumors (MC38 colorectal cancer cells) and administered with a murine version of MP0317 show an accumulation of the construct in the tumors		
			• A significant anti-tumor effect as well as an increased memory antitumor immunity was observed in the MC38-FAP mice administered with the murine version of MP0317		
			• No toxicity (measured by blood cytokines IL6, TNF-α, IFN-γ and IL12p70 and by the hepatotoxicity markers AST and ALT) was observed in these mice administered with the construct, as compared to the CD40-mAb		

In the early 2000s, MDX-210 (anti-HER-2 × anti-FcγRI) and MDX-H210 (humanized version of MDX-210) both entered several phase I and II clinical trials for the treatment of HER2^+^ breast, ovarian or prostate cancers, alone and in combination with other effectors, such as G-CSF and IFN-γ. In 2008, MDX-447 (anti-EGFR × anti-FcγRI) was evaluated in a phase I/II clinical trial for various EGFR^+^ tumors (including head and neck, kidney, bladder and prostate cancers). Unfortunately, despite a well-tolerated response in patients, clinical studies of all three engagers were discontinued due to a lack of efficacy and therefore a lack of significant antitumor responses in human patients [75].

Macrophages and neutrophils are also characterized by the expression of SIRPα, an inhibitory receptor that engages with CD47 (which is overexpressed in many tumor cells), leading to the “don’t-eat-me” signal, which is used by cancer cells to avoid destruction by myeloid cells. To circumvent this signal inhibition, Ring et al. reported the development of bispecific anti-SIRPα (which prevents the interaction with CD47 and therefore phagocytosis inhibition) coupled with an anti-CD70, a TAA associated with several cancers, such as non-Hodgkin lymphoma, multiple myeloma, renal cell carcinoma, and glioblastoma [78]. In this study, the authors were able to show a significant enhancement of phagocytosis against several cancer cell lines, as well as inhibition of Burkitt’s lymphoma cell growth in mouse models.

Another strategy consists of directly targeting CD47 (the ligand of SIRPα) to block access to the SIRPα receptor to ultimately enhance the efficacy of the molecule. Several studies have reported the development of bispecific antibodies directed against CD47 and a tumor-associated antigen (TAA), such as EGFR [79] or CD19 (for the treatment of B-cell lymphoma and leukemia) [80, 81]. Some authors have also developed anti-CD47 × anti-PDL1 constructs that can both prevent the signal inhibition mediated by the CD47/SIRPα pathway and inhibit PD-1/PD-L1 checkpoint inhibitor signaling [75]. Although these approaches are worth mentioning, they do not qualify as immune cell engagers since both targeting moieties are found on tumor cells.

FcαRI (CD89), another crucial receptor present on the surface of neutrophils and macrophages, is also the target of several bispecific myeloid cell engagers. One recent example is the anti-CD20 × anti-FcαRI compound developed by Li et al. in the context of B-cell malignancies and lung cancer [76]. The authors showed that anti-CD20 × FcαRI could effectively mediate the ADCC of Raji cells in vitro and stimulate the regression of Raji tumor cells in NOD/SCID mice in the presence of neutrophils. In a transgenic mouse model expressing human FcαRI only in monocytes and macrophages (FcαRI Tg mice), the administration of their bispecific antibody was further able to stimulate the regression of Lewis lung cancer cells transfected with human CD20 (LLC-hCD20), an effect not observed on WT mice, which indicates the necessity of FcαRI expression for treatment efficacy [76].

In an attempt to combine the advantages of targeting multiple sites, Kelton et al. reported the development of an “IgGA”, a hybrid antibody with the ability to bind both FcγR (such as IgG) and FcαR (such as IgA) [82]. These “cross-isotype” constructions, which were initially developed in combination with the anti-HER2 antibody trastuzumab but also described with anti-CD20, enhance the effector functions of myeloid cells (ADCC, ADCP and CDC) both in vitro and in vivo and could therefore represent a new area of research for improved myeloid cell immunotherapy [82–84].

Similarly, in 2019, Heemskerk et al. reported the development of “TrisomAbs”, a trispecific engager developed to stimulate myeloid cells via the FcαRI and FcγRI receptors [85]. Previous studies reported an increase in the antitumor activity of neutrophils in the presence of IgA (a ligand of the FcαRI receptor), suggesting that the engagement of this receptor might be beneficial for triggering the cytotoxicity of neutrophils to cancer cells [85]. By combining this targeting moiety and an anti-EGFR/anti-gp75 moiety with an Fc-based structure, TrisomAb was able to stimulate the recruitment and function (ADCC and ADCP) of neutrophils, macrophages and NK cells. This molecule ultimately promoted antitumoral effects in vitro on mice bearing B16F10gp75 melanoma cells, as well as on ex vivo neutrophils derived from colorectal cancer patients [85].

Macrophages are also involved in the immune response against HIV infections, notably through the use of sensor receptors that stimulate antiviral immunity [86]. In the late 1990s, the concept of macrophage engagers emerged in this field with the construct MDX-240, an anti-gp41 × anti-FcγRI bispecific antibody [87, 88]. Because MDX-240 can trigger cytotoxicity and reduce HIV infectivity in human monocyte-derived macrophages (MDMs) in vitro, this engager entered a clinical trial for late-stage AIDS patients in France and Belgium [89]. However, the results of this study were not found, and no publications were published after that period, highlighting the likely limited efficacy of this approach.

Compared to macrophages, neutrophils have a much more direct effect on anti-HIV immunity and possess direct antimicrobial mechanisms. Moreover, neutrophils seem to be closely related to HIV infections, as HIV-infected patients often develop neutropenia (a decreased count of peripheral neutrophils), as well as generally decreased neutrophil function. Moreover, some studies have reported a putative protective role of neutrophils against HIV infection, as evidenced by a negative correlation between HIV acquisition and the peripheral blood neutrophil count, although this result remains controversial [90]. Stimulation of the action of neutrophils toward HIV-infected cells by engagers seems therefore to be a worthy approach to investigate.

As such, Duval et al. designed an anti-CD89 (FcαRI) × anti-gp41 construct that promotes the in vitro destruction of HIV-infected cells by neutrophils [91]. Encouraging results were reported, as the bispecific structure could bind to primary isolates from all clades of HIV-1 and trigger neutrophil-mediated cytotoxicity against HIV-infected cells, although its potential use in clinics was hindered by difficulties in terms of large-scale production. The same team reported similar constructs but with modified structures in 2016 to solve this issue [92]. Instead of their initial chemical conjugation using Sulfo-SMCC cross-linkers, the authors remodeled their bispecific construct in the form of conventional linkers of scFv fragments. Surprisingly, although the “scFv form” was also able to bind to its targets, the constructs failed to trigger antibody-dependent cell-mediated viral inhibition (ADCVI) similar to that previously observed with chemical conjugates. Therefore, the construct was remodeled again to obtain a dimeric or tetrameric Fab-like structure, which could restore the initial ADCVI activity due to improved flexibility and therefore better bridging between the neutrophils and the HIV-infected cells [92]. Eventually, this approach could favor the action of neutrophils against HIV-infected cells, although no additional manuscripts were published. However, to the best of our knowledge, no other studies have since reported the development of neutrophil engagers in the context of cancer and HIV infections.

While this review focused on multispecific immune cell engagers for cancer and for viral diseases, interestingly, some studies have been able to bridge the gap between them. In a recently published paper from He et al., the authors addressed the challenge of Epstein‒Barr virus (EBV) infection, for which it is crucial to fight both the virus and the EBV^+^ tumor cells potentially resulting from this infection [93]. Indeed, EBV initially infects B lymphocytes through specific receptors (such as gp350/gp220), allowing the virus to enter these cells and to spread to other cell types, such as epithelial cells and occasionally T cells, NK cells and smooth muscle cells, resulting in oncogenesis. Ultimately, EBV infection leads to various blood and epithelial cancers. In their paper, He and collaborators designed an anti-gp350 × anti-CD89 bispecific antibody that can bind on the one hand to EBV and EBV^+^ tumor cells and on the other hand to CD89^+^ cells, i.e., myeloid cells. The authors reported the inhibition of EBV infection and decreased EBV^+^ B lymphoma cell growth, both in vitro and in NSG mice infected with the virus [93]. This novel approach could therefore overcome the limitations of current treatments, such as rituximab, which has focused only on the destruction of tumor cells and not viral particles and could ultimately lead to a complete cure for EBV infection.

Because macrophages and neutrophils have similar patterns of receptor expression, most engagers that have been developed to stimulate myeloid cells are targeted to both cell types. Although this lack of specificity does not pose a substantial issue, given the complementary nature of their effector functions, the challenge in developing myeloid cell engagers appears to lie in choosing the appropriate receptor to target. Indeed, as evidenced in Table 1, attempts to develop constructs targeting FcγRI receptors have been unsuccessful, and all clinical trials conducted for these molecules have failed. In contrast, targeting FcαRI, particularly in combination with FcγRI (either with a cross-isotype antibody or trispecific engagers), seems to be a promising avenue. Notably, however, that these constructs are only in the preclinical stage, and further studies and clinical trials are required to determine their efficacy and utility as immunotherapeutic treatments.

### Dendritic cells

Dendritic cells (DCs) represent important actors to mention. In the specific context of infection, these cells are able to recognize pathogens and initiate adaptive immune responses at a much greater potency than macrophages and monocytes [94]. After recognition of specific antigens and maturation, DCs migrate to secondary lymphoid tissues, where they interact with B and T lymphocytes and trigger a specific response via their major histocompatibility complex (MHC) [94].

Dendritic cells play important roles in the anticancer immune response. These roles range from the capture and processing of tumor antigens to migration to secondary lymphoid organs and presentation to naïve T cells, and the production of chemokines and cytokines to trigger T-cell recruitment and function [95]. Since the vast majority of DC functions rely on their interactions with T cells, DCs are interesting targets for immune cell engagers because enhancing their activity can indirectly improve T-cell priming and function while avoiding the systemic adverse events observed with “direct” T-cell engagers [96].

Similarly, Hägerbrand et al. recently reported a CD40 × TAA bispecific antibody targeting CD40 (a receptor expressed on the surface of DCs but also on the surface of B cells and macrophages) to enhance the upregulation of several maturation markers at the DC surface (CD80 and CD86) and to stimulate the production of proinflammatory cytokines (IL-12) [97]. The construct was designed as two alternatives with two different tumor-targeting moieties: one against either EpCAM or one against CEA. The engagement of CD40 with the construct was reported to stimulate the priming of T cells, as well as the activation of APCs, including DCs, at a higher rate than with an antibody targeting only CD40. Ultimately, the administration of their construct to hCD40tg mice bearing MB49-EpCAM- or CEA-transfected MC38 tumor cells led to significantly reduced tumor volumes and enhanced survival [97]. Preliminary studies using NHPs revealed good tolerance of the drug, with no adverse events related to cytokine or liver enzyme levels.

In addition to this study, the targeting of CD40 and TAAs with bispecific antibodies has been reported several times, as summarized in Table 1 [96, 98, 99]. The construct anti-CD40 × anti-MSLN (ABBV-428) has even entered a clinical trial and demonstrated an acceptable safety profile in a phase I trial in patients with advanced mesothelioma or ovarian cancer, although low clinical activity was observed (NCT02955251) [100].

Using a completely different approach, Sung et al. reported ABL501, an anti-LAG-3 × PD-L1 bispecific antibody that inhibits both the immunoregulatory receptors LAG-3 (expressed on the surface of DCs and T cells) and PD-L1 (expressed on the surface of tumor cells) [99]. Using this molecule, the authors aimed to improve the classical PD1/PD-L1 blockade therapy that has shown limited efficacy thus far due to the restricted scope of cancer to which it can be applied. In their study, they showed improved DC activation and priming of T cells, resulting in induced CD8^+^ T-cell activation and eventually greater cytotoxicity against the target. In humanized mouse tumor models, treatment with ABL501 increased the percentage of tumor-infiltrating lymphocytes (TILs) inside the tumor and, more importantly, increased survival and decreased tumor growth. No significant toxicity was observed in mice or in cynomolgus monkeys. These excellent results led to the launch of a clinical trial to evaluate the safety and tolerability of ABL501 in patients with progressive, unresectable or metastatic solid tumors (NCT05101109).

Other alternatives to CD40 bispecific engagers include the designed ankyrin repeat protein (DARPin) anti-FAP × anti-CD40 (MP0317), which targets cancer-associated fibroblasts [101], and immune-stimulator antibody conjugates, for which the targeting activity is complemented with the delivery of immunostimulatory agents specific for innate immune receptors, as recently reviewed [5].

In conclusion, although DCs are often considered indirect actors in antitumor responses, they play crucial roles in T-cell activation and modulation of the immune response against cancer cells. Although the number of reported DC engagers is limited thus far, the results obtained from the few preclinical studies look promising and have already resulted in two clinical trials, indicating that CD40 bispecific antibodies will likely expand in the next few years and become additional assets to enhance antitumor immunotherapies.

## Conclusions and perspectives

Driven by promising results from preclinical and clinical studies, multispecific engagers are becoming important new agents in immunotherapy. Starting from bispecific T-cell engagers, these constructs were complexified to increase the number of targeting moieties, to diversify the targets, and to enhance their effects.

The remarkable functional efficacy exhibited by T-cell engagers has resulted in marketing authorizations for several of them, including blinatumomab, and numerous others are currently undergoing evaluation by regulatory authorities. Nonetheless, T-cell engagers are also associated with a concerning safety profile, as some patients undergoing this therapy have faced serious toxicity issues, limiting their use in clinical practice. Because of these limitations, interest in other cell types, such as NK cells, which harbor multiple antitumor activities, has increased.

NK immunotherapies may overcome the current limitations of T-cell-based approaches for many reasons, as detailed in the present review. Nevertheless, in HIV or CMV infection, the CD56^neg^ NK cell subset, which harbors impaired cytotoxic function due to the expression of a wide range of inhibitory receptors, is expanding [102]. Therapeutic strategies harnessing NK cells with NK-cell engagers should carefully address these hurdles for ongoing research and development and propose alternatives, such as the administration of exogenous NK cells with high cytotoxic potential or the stimulation of memory-like T cells or NK cells, to be as effective as T-cell therapies. The ever-growing knowledge of the “trained immunity” field provides promise for understanding the pivotal epigenetic modifications leading to long-lived memory-like NK cells [103] and trained myeloid cells [104, 105] that could further sustain adaptive and antibody responses to overcome all these challenges.

Engagers for cellular cancer therapy should pave the way for the treatment of viral infections, although many specific challenges encountered in viral infections still need to be considered. While engagers have been engineered to specifically target HIV-infected cells, they might likewise target healthy cells expressing similar antigens. Therefore, the ultimate goal is to target a specific marker of the long-lived HIV latent reservoir. A number of cellular HIV-1 reservoir markers have been proposed in recent years, such as HLA-DR, PD-1, TIGIT, and CD32a, which lack specificity, and more recently, markers of novel axes, such as the hypoxia-CD73-adenosine axis, which needs further investigation [106]. Another major challenge associated with engager therapy for HIV infection is immune escape resulting from mutations in viral proteins and its low and versatile expression during chronic infection [11]. Therefore, identifying future engagers with multiple highly conserved sites on gp160 or with two or three gp120/gp41 domains is crucial.

Persistent barriers linked to the immunosuppressive effect of viral infection or the tumor microenvironment underscore the need for continued research. In this regard, the clear contribution of TGF-β signaling to T-cell and NK-cell dysfunction warrants further development of engagers and the use of TGF-β inhibitors as potential therapeutics for treating both cancer and viral infections [107, 108].

Finally, another major issue remains the delivery of the engagers to the anatomical or cellular sites of the viral reservoirs or tumors. The small size of engagers is therefore crucial compared to that of Ab fragments and Ab fusion proteins for penetrating normal tissues, including lymphoid tissue for HIV-1 (in which the virus mainly replicates) or malignant tissues for solid tumors, which harbor a hostile microenvironment composed of dense stroma and extracellular matrix constituents. Arming engagers with chemokines to attract T cells or NK cells near the tumor should therefore be considered.

Immunotherapies for viral diseases currently lag behind those for cancer, and immunotherapeutic interventions for HIV or HBV must not compromise the safety and efficacy of existing drug regimens. Encouraging progress has been achieved to overcome this pivotal barrier to scale-up and translate such strategies into preclinical or clinical trial stages for successful treatment. The further development of more targeted and combined approaches is likely to reach the goal of a functional cure. A tempting speculation is that the combination of T-cell and NK/myeloid-cell engagers might be a more successful option, although it would be more costly.
